# Acute and longer-term psychological distress associated with testing positive for COVID-19: longitudinal evidence from a population-based study of US adults

**DOI:** 10.1017/S003329172100324X

**Published:** 2021-07-26

**Authors:** Michael Daly, Eric Robinson

**Affiliations:** 1 Department of Psychology, Maynooth University, Co. Kildare, Ireland; 2 Institute of Population Health Sciences, University of Liverpool, Liverpool, UK

**Keywords:** COVID-19, coronavirus infection, mental health, psychological distress, longitudinal research, nationally representative study

## Abstract

**Background:**

The novel coronavirus (SARS-CoV-2) has produced a considerable public health burden but the impact that contracting the disease has on mental health is unclear. In this observational population-based cohort study, we examined longitudinal changes in psychological distress associated with testing positive for coronavirus disease 2019 (COVID-19).

**Methods:**

Participants (*N* = 8002; observations = 139 035) were drawn from 23 waves of the Understanding America Study, a nationally representative probability-based online panel of American adults followed-up every 2 weeks from 1 April 2020 to 15 February 2021. Psychological distress was assessed using the standardized total score on the Patient Health Questionnaire-4.

**Results:**

Over the course of the study, 576 participants reported testing positive for COVID-19. Using regression analysis including individual and time-fixed effects we found that psychological distress increased by 0.29 standard deviations (*p* < 0.001) during the 2-week period when participants first tested positive for COVID-19. Distress levels remained significantly elevated (*d* = 0.16, *p* < 0.01) for a further 2 weeks, before returning to baseline levels. Coronavirus symptom severity explained changes in distress attributable to COVID-19, whereby distress was more pronounced among those whose symptoms were more severe and were slower to subside.

**Conclusions:**

This study indicates that testing positive for COVID-19 is associated with an initial increase in psychological distress that diminishes quickly as symptoms subside. Although COVID-19 may not produce lasting psychological distress among the majority of the general population it remains possible that a minority may suffer longer-term mental health consequences.

## Introduction

The coronavirus disease 2019 (COVID-19) pandemic radically changed daily life for much of the world's population and by July 2021 had led to 194 million confirmed infections worldwide including almost 35 million in the United States (WHO, 2021). Although there has been a surge in research examining the potential population mental health consequences of living through the pandemic (Robinson, Sutin, Daly, & Jones, 2021; Salari et al., 2020), few studies have specifically examined the mental health impact of contracting the novel coronavirus (SARS-CoV-2). A review of studies examining severe acute respiratory syndrome (SARS) and Middle East respiratory syndrome (MERS) concluded that severe coronavirus infections are linked to elevated depression and anxiety disorder in the months following infection (Rogers et al., 2020). In the context of COVID-19, there is growing concern that the disease may lead to prolonged effects after recovery including headaches, muscle and body ache, and persistent tiredness (Sudre et al., 2021) and that these post-acute COVID-19 symptoms (often termed ‘long COVID’) may result in prolonged mental health problems (Del Rio, Collins, & Malani, 2020). However, there is currently a dearth of evidence on the mental health impacts of contracting COVID-19 and the current study aimed to address this gap by examining longitudinal data on US adults to estimate changes in psychological distress in response to contracting COVID-19.

Research to date suggests that reporting a positive test or symptoms consistent with COVID-19 is associated with raised anxiety and depression levels in cross-sectional samples of the general population in the UK and Ireland (Hyland et al., 2020; Shevlin et al., 2020). Similarly, reporting symptoms compatible with COVID-19 has been linked to worse mental health in a large-scale study (*N* = 69 054) of university students in France (Wathelet et al., 2020). A large-scale cross-sectional study of UK adults (*N* = 44 775) has shown that reporting a diagnosis of COVID-19 is associated with raised levels of self-harm and suicidal ideation (Iob, Steptoe, & Fancourt, 2020). Although these studies link potential COVID symptoms or diagnosis with increased mental health problems, the cross-sectional nature of study means that it is plausible that findings are explained by confounding bias or reverse causality. For example, individuals with existing mental health problems may be more likely to report experiencing COVID-19 symptoms or to contract COVID-19 (van der Meer et al., 2020).

In line with this, a large-scale record linkage study of 61 million US adults found that those with a recent diagnosis of a mental disorder were at an increased risk of COVID-19 infection (Wang, Xu, & Volkow, 2021b). This finding was confirmed by a subsequent study of the health records of almost 70 million patients in the United States (Taquet, Luciano, Geddes, & Harrison, 2021b). Within the same study, 62 354 patients contracted COVID-19 between January and August 2020 and this group was at an increased risk of first-time diagnosis of psychiatric disorder (particularly anxiety disorder and insomnia), within 3-months relative to those experiencing other health events such as influenza (Taquet, Geddes, Husain, Luciano, & Harrison, 2021a; Taquet et al., 2021b). This finding suggests that contracting COVID-19 may increase the risk of mental health problems.

However, in the absence of longitudinal data on mental health symptoms prior to infection and data on the background characteristics of patients, it is unclear whether this association could be attributed to pre-illness factors, such as subclinical mental health symptoms or confounding lifestyle factors that predispose individuals toward more severe COVID-19 outcomes and poorer mental health (Popkin et al., 2020; Wang, Kaelber, Xu, & Volkow, 2021a). It is also not clear whether the increased risk of psychiatric problems identified may be representative of the impact of COVID-19 in the general population. For instance, a follow-up study drawing on the same health records showed that the onset of mood and anxiety disorders at 6 months was increased chiefly among a subset of COVID-19 patients experiencing brain dysfunction as a result of severe infection (Taquet et al., 2021a).

To understand the potential impact of COVID-19 infection on mental health in the general population, there is a need for representative longitudinal data which assess whether pre-COVID-19 mental health symptoms change sequentially as a result of infection. The objective of the current study was, therefore, to examine longitudinal changes in psychological distress both during infection and after recovery in large nationally representative cohort of US adults. We also examined if results were consistent across population demographics and the extent to which changes in psychological distress were explained by the severity and duration of COVID-19 symptoms (e.g. respiratory difficulties) experienced as a result of infection.

## Methods

### Sample

Participants were drawn from the Understanding America Study (UAS), a nationally representative probability-based online panel of 9063 individuals (Alattar, Messel, & Rogofsky, 2018; Kapteyn et al., 2020). The sample is comprised of non-institutionalized civilian adults aged 18 years and over. Participants were recruited in batches, first via a random sample of addresses identified via the ASDE Survey Sampler (http://surveysampler.com/) and then addresses were drawn from the US Postal Service Computerized Delivery Sequence file to refresh the study sample (Alattar et al., 2018). In this second stage, household addresses were selected at random from zip codes identified via an adaptive sampling algorithm that rebalances the sample toward the demographic composition of the US population (Kapteyn et al., 2020). The UAS has an estimated cumulative weighted recruitment rate of 13–15% (Kapteyn et al., 2020). Surveys are administered via the internet and eligible participants without computers or internet access are provided with internet-connected tablets.

From 1 April 2020, respondents were invited to take part in a continuous tracking study where participants completed surveys every 2 weeks during the COVID-19 pandemic. The survey has been used to examine changes in mental health (Daly & Robinson, 2020) and COVID-19-related perceptions and protective behaviors (Daly, Jones, & Robinson, 2021; Robinson & Daly, 2020).

From a total number of 8129 participants and 142 573 observations across 23 waves of data collection we excluded 127 participants and 1475 observations due to missing data on demographic characteristics. A further 102 observations were dropped due to missing COVID-19 test or symptom data and 1961 observations due to missing data of Patient Health Questionnaire-4 (PHQ-4). We utilize data from the remaining 8002 UAS panel members who provided a total of 139 035 observations from 1 April 2020 to 15 February 2021. The average number of participants in each survey wave was 6045 and participants completed 17.4 out of 23 possible surveys on average. The dates of data collection and number of participants in each survey wave can be seen in online Supplementary Table S1.

Further details of the UAS survey administration and sampling methodology can found elsewhere (Alattar et al., 2018; Angrisani, Kapteyn, Meijer, & Saw, 2019; Kapteyn et al., 2020). The UAS was approved by the University of Southern California human subjects committee Internal Review Board (IRB) and informed consent was obtained from all participants.

### Measures

#### Psychological distress

Psychological distress was assessed using the validated four-item PHQ-4, which has been shown to be reliable and responsive to changes in mental health (Kroenke, Baye, & Lourens, 2019; Kroenke, Spitzer, Williams, & Löwe, 2009; Löwe et al., 2010). The scale is comprised of two items from the PHQ-9 and two items from the Generalized Anxiety Disorder-7 (GAD-7). The items selected assess core symptoms of anxiety (i.e. ‘feeling nervous, anxious, or on edge’ and ‘not being able to stop or control worrying’) and depression (i.e. ‘little interest of pleasure in doing things’ and ‘feeling down, depressed, or hopeless’). Participants were asked how often over the last 2 weeks they have been bothered by these problems and reported their answers on a four-point scale with response options *not at all* = 0, *several days* = 1, *more than half of days* = 2, and *nearly every day* = 3. Total scores on the scale range from 0 to 12 with higher scores indicating greater distress (mean Cronbach's *α* = 0.92, range = 0.88–0.93). Total PHQ-4 scores were standardized to have a mean of zero and standard deviation (s.d.) of 1.

#### COVID-19 testing

As part of each survey wave, participants reported whether they had been tested for coronavirus since they last completed the UAS continuous tracking survey and were reminded of the date of their most recent survey. Participants reported the result of the coronavirus test using four options: (1) ‘I have been tested and I tested positive (I had coronavirus)’, (2) ‘I have been tested and I tested negative (I did not have coronavirus)’, (3) ‘I have been tested and I do not know the result’, and (4) ‘I have not been tested’. Those who tested positive were classified as having COVID-19 and others were classified as not testing positive. Where participants reported testing positive in more than one survey wave (typically in waves directly following the first positive COVID-19 test), we examined the first positive test only.

#### COVID-19 symptoms

In each survey wave, participants indicated whether or not they had experienced common symptoms of COVID-19 in the past 7 days. In line with a recent large-scale assessment of the clinical spectrum of COVID-19 symptoms (Eythorsson et al., 2020) we included assessments of upper respiratory (i.e. runny or stuffy nose, sore throat, sneezing, and lost sense of smell), lower respiratory (i.e. chest congestion, cough, and shortness of breath), gastrointestinal (i.e. vomiting, diarrhea, and abdominal discomfort), and generalized symptoms (i.e. fever or chills or body temperature higher than 100.4 F or 38.0°C, headache, muscle, or body aches). The percentage of all symptoms reported in the past 7 days was examined as our primary indicator of participant experiences of COVID-19 (ranging from 0 = no symptoms experienced, to 100 = all symptoms experienced). The composite score of the percentage of symptoms reported demonstrated adequate reliability across survey waves (mean Cronbach's *α* = 0.76, range: 0.72–0.79).

### Demographic characteristics and other potential moderating factors

We examined a set of core demographic factors that may moderate the association between COVID-19 and psychological distress: participant age (18–39, 40–59, 60+ years), gender (male, female), race/ethnicity (White, Hispanic, Black, Other race/ethnicity), and annual household income levels (total income of family members residing in the home including from jobs, businesses, rent, pension, Social Security payments, and other sources) was divided approximately into tertiles (⩽$40 000, $40 000–100 000, ⩾$100 000 per annum). In addition, we tested whether the potential association between COVID-19 and distress levels was moderated by the presence of a pre-existing diagnosis of a chronic physical health condition (i.e. diabetes, cancer, heart disease, kidney disease, asthma, chronic lung disease, and autoimmune disease) or a mental health condition (i.e. anxiety disorder, attention deficit hyperactivity disorder, bipolar disorder, eating disorders, depressive disorders, obsessive compulsive disorder, posttraumatic stress disorder, or schizophrenia/psychotic disorder) assessed via a self-report check-list of conditions.

Finally, we tested whether a series of additional factors may moderate the association between COVID-19 and distress: number of household members, presence of child in the household, presence of parents in the household, whether or not the participant reports having health insurance, substance use (mean number of days in the past week participant used cannabis, recreational drugs, smoked, and drank alcohol), perceived risk of job loss or running out of money in the next 3 months (percent chance from 0 to 100 for each), social support from friends and family [number of days in the past week connected socially with friends or family (online or in-person)], and resilience. Resilience was assessed at the beginning of May 2020 using the six-item Brief Resilience Scale (BRS) (Smith et al., 2008). Participant responses were recorded on a 5-point Likert scale (‘strongly disagree’ to ‘strongly agree’) and the scale demonstrated adequate reliability levels (Cronbach's *α* = 0.86).

### Statistical analysis

All analyses were carried out in Stata version 17 using survey weights to generate nationally representative estimates. In the UAS sampling weights adjust for differential probabilities of selection into the UAS and post-stratification is used to adjust the weights so that each survey matches the distribution of demographic characteristics of the US population. This poststratification stage aligns the demographic composition of each survey wave sample with 13 population characteristics (e.g. age, gender, race/ethnicity, education, household income levels, and the geographic distribution of the population) using Continuous Population Survey estimates from the Annual Social and Economic Supplement. The use of poststratification ensures that the sample remains nationally representative over time despite the presence of missing data due to nonresponse. Weighted and unweighted sample characteristics at the start and end of the survey period are shown in online Supplementary Table S2. Further details of the UAS weighting methodology can be found in Angrisani et al. (2019).

In preliminary analyses, to test the validity of the self-reported COVID-19 positive test data we compared the estimated population prevalence of positive tests in each wave of the UAS and in the US adult population over the same 2-week periods from 1 April 2020 to 15 February 2021. Total case numbers per day were drawn from the Centers for Disease Control and Prevention COVID Data Tracker (CDC, 2021) and an adjustment was applied to correct for the presence of those under 18 in the daily case figures (see online Supplementary materials – Section 1). We also compared the prevalence of each COVID-19 symptom examined in this study with the symptoms identified in two large-scale studies examining the symptoms of those with confirmed positive COVID-19 tests (Eythorsson et al., 2020; Wohl et al., 2021). Finally, we tested whether testing positive for COVID-19 was linked to subsequent drop-out or a reduced level of participation in the UAS continuous tracking survey.

Our main analysis used fixed-effects regression to estimate the link between reported coronavirus disease and changes in psychological distress within the same individuals across multiple time-points. The basic fixed-effects model estimating the association between testing positive for COVID-19 (*β*_1_*COVID_it_*) and psychological distress (*PHQ_it_*) experienced by individuals *i* across survey waves *t* can be expressed as:1
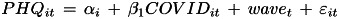


The inclusion of individual fixed effects (*α_i_*) provides a control for all stable individual characteristics that could influence susceptibility to COVID-19 or psychological distress (e.g. gender, race, and family background). Although individual fixed effects adjust for all unchanging characteristics that could impact this relationship potential interactions between stable characteristics are not accounted for. Furthermore, it remains possible that the link between COVID-19 and distress could be impacted by seasonal and other calendar effects associated with the pandemic (e.g. changing macroeconomic conditions). For this reason, we also included survey wave fixed effects (*wave_t_*) for each 2-week period during which the study surveys were completed. By including individual and time-fixed effects, we eliminated bias from all factors that are fixed over time for individuals and factors that vary over time but are constant across individuals.

The fixed-effects approach employed in this study compares the distress levels of an individual when they have tested positive for COVID-19 (and in waves before/after testing positive where examined) with their distress level in other waves and pools this information across the sample. In this way, the increase in distress associated with testing positive for COVID-19 can be estimated relative to a ‘baseline’ level (Clark, Diener, Georgellis, & Lucas, 2008), which is the predicted level of distress at other time-points experienced by those who at some stage test positive for COVID-19. For modeling purposes, the sample included both those who did and did not test positive for COVID-19 over the course of the study. We also adopted this approach to examine changes in mental health experienced by those who went for a COVID-19 test and tested negative for the disease.

Lags and leads in the effect of COVID-19 were examined to identify whether distress increased before or after testing positive for the disease. Lead effects identified whether distress increased from baseline levels in advance of a positive test. Lag effects identified whether there was a residual effect of COVID-19 on psychological distress in subsequent survey waves after the participant tested positive for the disease. Lag and lead analyses relied on survey waves completed immediately before and after the wave where participants tested positive for COVID-19. In addition, we tested whether the association between COVID-19 and psychological distress was moderated by participant sociodemographic characteristics, resilience, and the presence of chronic physical or mental health conditions.

Finally, we utilized the fixed-effects model outlined above to examine the association between testing positive for COVID-19 and experiencing symptoms of the disease, and the association between COVID-19 symptoms and psychological distress. Drawing on this information we conducted mediation analysis using the *khb* command in Stata to estimate the indirect effect of COVID-19 on psychological distress via COVID-19 symptoms (Karlson, Holm, & Breen, 2012).

## Results

### Sample characteristics

The demographic characteristics and symptoms experienced by those who tested positive for COVID-19 and the remaining UAS participants are shown in Table 1. Those who tested positive were more likely to be aged 40–59 years compared to the remainder of the sample. Those reporting a positive test were also more likely to be of Hispanic race/ethnicity. The percentage of participants who took part in the next survey wave immediately following a positive COVID-19 test (82.6%) was in line with the overall response rate to surveys in this study (75.5%). In addition, we found that those who tested positive for COVID-19 did not complete a significantly different number of surveys (*M* = 17.85 waves completed, s.d. = 6.5) to participants who did not test positive for COVID-19 (*M* = 17.34 waves completed, s.d. = 7.24) (*B* = 0.51, s.e. = 0.31, *p* = 0.10). Those who tested positive for COVID-19 at any stage in 2020 (*N* = 426) were significantly more likely compared to other participants to take part in the UAS survey in 2021 [odds ratio = 1.82, (95% confidence interval (CI) 1.28–2.61), *p* < 0.01], although retention was very high for both groups (92% *v.* 86.3%).

**Table 1.** Demographic characteristics and symptoms experienced by participants reporting a positive COVID-19 test (*N* = 576) and remaining participants (*N* = 7426) in the Understanding America Study

	COVID-19 reported^a^ (*N* = 576)	No COVID-19 reported^b^ (*N* = 7426)
	%	%
Age group, years		
18–39	38.8	42.9
40–59	39.4	30.6**
60+	21.8	26.5
Female	51.5	51.9
Hispanic	25.8	17.6**
Black	9.1	12.2
Other race/ethnicity	4.6	6.1
White	60.5	64.2
Low income (⩽$40 000)	34.7	38.4
Medium income ($40 000–100 000)	46.3	39.8*
High income (⩾$100 000 per annum)	19.0	21.8
Physical health condition reported	36.7	32.1
Mental health condition reported	32.8	29.6
Symptoms reported in past 7 days		
Fever/raised temperature	41.8	1.7***
Headache	58.9	16.3***
Muscle or body ache	52.6	13.9***
Runny or stuffy nose	53.2	18.0***
Sore throat	31.9	5.0***
Sneezing	41.3	17.8***
Lost sense of smell	40.6	0.9***
Chest congestion	32.4	3.5***
Cough	59.0	9.9***
Shortness of breath	37.0	5.5***
Vomiting	9.2	0.9***
Diarrhea	31.1	5.5***
Abdominal discomfort	24.8	6.3***

*Note:* Weighted values are reported. Binary logistic regression analyses were used to test for differences between those reporting *v*. not reporting COVID-19.

a Symptoms are assessed during the wave where positive COVID-19 test was reported (*N* = 576).

b Symptoms are assessed across all available survey waves (*N* = 7426).

*p < 0.05, **p < 0.01, ***p < 0.001.

### COVID-19 positive tests

Between 1 April 2020 and 15 February 2021, 576 participants from the UAS sample reported that they had tested positive for COVID-19. On average, 39.5% of symptoms were experienced in the past 7 days among those testing positive for COVID-19. The most common reported symptoms among those who tested positive were cough (59%), headache (58.9%), nose congestion (53.2%), muscle or body aches (52.6%), fever or high temperature (41.8%), and loss of smell (40.6%), as shown in Table 1. The prevalence of COVID-19 symptoms identified in the current study was comparable to that identified in high-quality studies where a positive COVID-19 test was confirmed via laboratory tests, as shown in online Supplementary Table S3.

The total estimated prevalence of COVID-19 across all survey waves of the UAS examined was 9.39% and the estimated prevalence of the disease in the US adult population over the same period was 9.42%. For further details of how prevalence estimates were derived see Section 1 of the online Supplementary materials. There was also a high degree of overlap in the time trend of COVID-19 cases in the UAS sample and the US adult population, as can be seen in online Supplementary Fig. S1. The prevalence of COVID-19 across all UAS survey waves correlated strongly with the prevalence in the US population over the same 2-week periods (*r*(21) = 0.93, *p* < 0.001), as shown in online Supplementary Figs S2 and S3.

Of those who tested positive for COVID-19, 26% reported attending a healthcare facility or hospital in the past week during the survey wave where they tested positive for COVID-19.

### Fixed-effects regression analysis

**Fig. 1.**
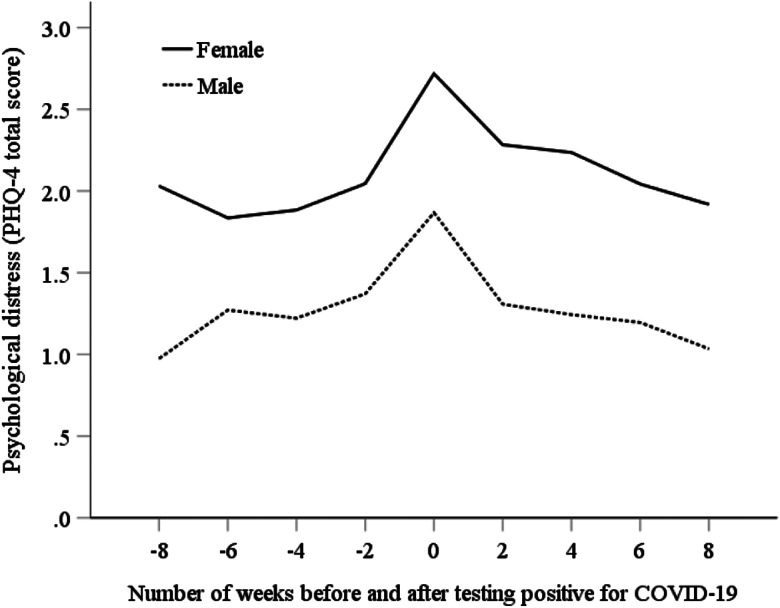
Trends in psychological distress assessed using the PHQ-4 (range = 0–12) in the weeks before and after testing positive for COVID-19.

An initial examination of the descriptive trends in psychological distress tracked from before to after reporting a positive COVID-19 test indicted that distress rose and peaked in the wave when a positive COVID-19 test was initially reported and declined in the weeks that followed. This trend was observed in males and females, as illustrated in the unadjusted trends presented in Fig. 1.

We found that testing positive for COVID-19 was associated with a 0.29 s.d. increase in psychological distress in the same survey wave (*B* = 0.29, s.e. = 0.04, *p* < 0.001). This increase reflected a change from a predicted level of distress of −0.03 (95% CI −0.03 to −0.02) in non-COVID-19 waves to 0.26 (95% CI 0.18–0.35) within the survey wave where participants tested positive for COVID-19. Those with COVID-19 who attended a healthcare facility or hospital in the previous week did not experience a significantly larger increase in distress (*B* = 0.31, s.e. = 0.08, *p* < 0.001) compared to those with COVID-19 who did not attend such facilities (*B* = 0.28, s.e. = 0.05, *p* < 0.001).

In contrast, those who tested negative for COVID-19 experienced a very small 0.03 s.d. increase in distress (*B* = 0.03, s.e. = 0.01, *p* < 0.01). Lead effects of testing positive for coronavirus were non-significant indicating that distress did not increase substantially in advance of testing positive, as shown in Table 2. In contrast, there was evidence for a lag effect where testing positive for COVID-19 was associated with a 0.16 s.d. increase in distress in the next survey wave (*B* = 0.16, s.e. = 0.06, *p* < 0.01) 2-weeks later. There was no evidence of significant associations between COVID-19 and distress beyond 2 weeks (lags of up to 8 weeks/four survey waves tested). Furthermore, the association between testing positive for COVID-19 and psychological distress was not moderated by sociodemographic factors, resilience, or the presence of mental health conditions (see online Supplementary Tables S4 and S5).

**Table 2.** Fixed-effects regression estimates of the association between testing positive for COVID-19 and changes in reported symptoms and psychological distress in the Understanding America Study

Outcome	Symptoms (%)			Psychological distress (*z*-score)		
*N*	8002	7199	6594	8002	7199	6594
Obs.	139 035	110 980	92 747	139 035	110 980	92 747
	*B* [95% CI]	*B* [95% CI]	*B* [95% CI]	*B* [95% CI]	*B* [95% CI]	*B* [95% CI]
COVID-19 in current wave	31.19*** [28.20–34.18]	31.78*** [28.27–35.30]	31.74*** [27.84–35.64]	0.29*** [0.21–0.37]	0.28*** [0.18–0.37]	0.27*** [0.17–0.37]
Two weeks prior to COVID-19 (*one wave lead effect*)^a^		8.48*** [5.80–11.17]	8.97*** [5.86–12.08]		0.03 [−0.06 to 0.12]	0.07 [−0.03 to 0.16]
COVID-19 2 weeks ago (*one wave lag effect*)^a^		8.72*** [6.03–11.41]	9.14*** [6.17–12.11]		0.16** [0.04–0.27]	0.17* [0.04–0.29]
Four weeks prior to COVID-19 (*two wave lead effect*)^a^			−0.70 [−2.36 to 0.83]			−0.02 [−0.09 to 0.05]
COVID-19 4 weeks ago (*two wave lag effect*)^a^			2.05 [−0.95 to 5.06]			0.09 [−0.03 to 0.22]

a Association between COVID-19 and distress among those completing surveys immediately before and after reporting a positive test.

*p < 0.05, **p < 0.01, ***p < 0.001.

Testing positive for COVID-19 was associated with a 31.2 percentage point (*p* < 0.001) increase in the percentage of COVID-19 symptoms from 8.1% (non-COVID-19 waves) to 39.3% (COVID-19 wave), as shown in Table 2. The percentage of symptoms reported increased by 8.5% (*p* < 0.001) from baseline levels in the 2-week period prior to testing positive for COVID-19 and remained 8.7% (*p* < 0.001) above baseline levels in the 2-week period after testing positive for the disease. There was no evidence of increases in symptom prevalence more than 2 weeks before or after the survey wave where the participant reported testing positive for COVID-19 (lags and leads of up to 8 weeks/four survey waves tested). Those with COVID-19 who attended a healthcare facility or hospital in the previous week experienced 13.9% (*p* < 0.001 for difference) more symptoms than those with COVID-19 who did not attend such facilities (49.5% *v.* 35.6% of symptoms reported).

### Mediation analysis

In our mediation model, the indirect effect of COVID-19 symptoms (*B* = 0.15, s.e. = 0.01, *p* < 0.001) accounted for 52% of the association between testing positive for the disease and the increase in psychological distress (see Table 3). The direct effect of testing positive remained significant after adjustment for symptom levels (*B* = 0.14, s.e. = 0.04, *p* < 0.01). The indirect effect of symptoms (*B* = 0.08, s.e. = 0.02, *p* < 0.001) accounted for over half (52.8%) of the lagged effect of COVID-19 on distress 2 weeks later, and after accounting for symptom levels the relationship between infection and lagged increase in distress was non-significant.

**Table 3.** Role of COVID-19 symptoms as mediators of the association between testing positive for COVID-19 and psychological distress in the United States

	Psychological distress (*z*-score)			
Timing of positive COVID-19 test	Current wave		Previous wave	
	*B*	s.e.	*B*	s.e.
Total effect of COVID-19^a^	0.29***	0.04	0.16**	0.06
Direct effect of COVID-19^b^	0.14**	0.04	0.07	0.06
Indirect effect via COVID-19 symptoms^c^	0.15***	0.01	0.08***	0.02

*Note:* Psychological distress is standardized to have a mean of zero and s.d. of 1.

a Total increase in psychological distress associated with testing positive for COVID-19.

b Increase in psychological distress associated with testing positive for COVID-19 not explained by COVID-19 symptoms.

c Increase in psychological distress associated with testing positive for COVID-19 explained by COVID-19 symptoms.

*p *<* 0.05, **p *<* 0.01, ***p *<* 0.001.

## Discussion

In this longitudinal population-based study, we used 23 biweekly waves of nationally representative longitudinal data to uncover evidence of changes in psychological distress in response to testing positive for COVID-19. Over the course of almost a full year of the pandemic 576 participants reported a positive COVID-19. We found that testing positive for COVID-19 was associated with an acute increase in distress levels (0.29 s.d. increase) after accounting for time-invariant unobserved heterogeneity using a fixed-effects model. This spike in distress declined substantially within 2 weeks and was not significantly different to baseline levels after this point. The increase and then partial recovery in psychological distress after 2 weeks did not vary significantly based on sociodemographic characteristics, participant resilience levels, or the presence/absence of pre-existing health conditions. Findings are consistent with proposals that those who contract COVID-19 may be at a greater risk of increased mental health symptoms compared to others living through the pandemic but not directly affected (Aknin et al., 2021). However, consistent with studies on mental health in the general population during the pandemic (Prati & Mancini, 2021; Robinson et al., 2021; Robinson & Daly, 2020), the present findings indicate that increases in distress attributable to infection are likely to be relatively transient and short-lived. Even after contracting a potentially deadly virus, the majority of the population appears to show resilience in mental health (van der Velden et al., 2021).

Because there have been concerns about the mental health consequences and long-term persistence of COVID-19 symptoms (e.g. Del Rio et al., 2020; Mahase, 2020) we examined changes in symptoms as a potential explanation for raised distress levels. In line with the overall results, symptoms increased during the wave of data collection when a positive COVID-19 test was first reported and declined rapidly thereafter. Furthermore, those experiencing a greater number of COVID-19 symptoms showed the largest increases in distress and COVID-19 symptoms explained over half of the prospective association between testing positive for COVID-19 and experiencing raised distress.

The short-term increase in distress observed may be explained by the likely combination of physical symptoms (i.e. the direct effects of ill health on mental health) and initial worry over health having tested positive, the latter which presumably dissipates once symptoms have subsided (Taquet et al., 2021a, 2021b). Overall, our findings are consistent with cross-sectional studies linking COVID-19 symptoms to measures of mental health (Hyland et al., 2020; Wathelet et al., 2020). However, unlike previous research which relied on between-person estimates, the present research confirms that within-person changes in distress occur in response to a self-reported positive test for COVID-19. In the current study, once COVID-19 symptoms had dissipated at around 2 weeks from reporting a positive test, levels of distress returned to baseline levels. Although we found no evidence of long-term mental health consequences of COVID-19 in the current study, recent findings suggest that if a severe infection occurs (Taquet et al., 2021a, 2021b) or if physical symptoms are maintained over time (i.e. ‘long COVID’), there may be elevated levels of distress in this minority of the population (Del Rio et al., 2020; Sudre et al., 2021).

A key strength of the current study was the repeated longitudinal assessment which allowed the point at which a positive COVID-19 test was reported and associated changes in distress to be identified within a narrow time window in a large nationally representative sample of US adults. However, this study is limited in several respects. Although the UAS utilizes probability-based sampling and has a high response rate for individual surveys, the initial recruitment rate was lower than high-quality traditional surveys which may limit the extent to which the sample represents the underlying population. We relied on participant reports of a positive COVID-19 test rather than verified test results and self-reports are known to be vulnerable to bias. However, we conducted a number of analyses to estimate the likely accuracy of self-reports. First, the prevalence of COVID-19 symptoms among those reporting having tested positive for COVID-19 in the current study was comparable to that from large-scale studies that have used both self-report and objective verification of infection (Eythorsson et al., 2020; Wohl et al., 2021). The estimated cumulative prevalence of COVID-19 cases in the US population and the UAS sample were closely aligned (9.4% in each) and we observed strong concordance between the time trend of positive cases in the population and the UAS sample. In addition, we found that those who sought a COVID-19 test (presumably due to concerns about being infected) but tested negative showed a minimal adverse change in their distress levels, which indicates that findings are unlikely to be explained by general concerns or worries about health as opposed to an actual COVID-19 infection. We also found no evidence that testing positive for COVID-19-predicted increased attrition or number of surveys completed in the current study. This suggests that bias in our assessment of confirmed cases may have been minimal.

Although we do not find evidence that testing positive for COVID-19 increases risk of dropout, it is likely that participants with the most severe infections were not included in the current study. A subgroup of those testing positive for COVID-19 indicated they attended a healthcare facility or hospital, but this group did not experience a larger increase in distress than those not attending such facilities. Recent evidence suggest that an increased risk of common psychiatric disorders may be experienced chiefly by a subset of hospitalized patients experiencing encephalopathy (brain disorder, disease, or dysfunction) as a result of severe COVID-19 infection (Taquet et al., 2021a, 2021b; Taylor, Landry, Paluszek, Rachor, & Asmundson, 2020). Therefore, the present findings provide information on the mental health consequences of testing positive for COVID-19 among the overall general population and not among those who developed COVID-19 and become critically ill or have suffered major adverse effects. It will, therefore, be important to continue to monitor long-term mental health outcomes at the population level and among those experiencing severe COVID-19 infections.

We examined a range of population demographics, including sub-groups who are at an increased risk of becoming seriously ill after testing positive for COVID-19 (e.g. pre-existing physical health conditions and ethnic minorities) but found no evidence that changes in distress differed across demographics. Yet, although we utilized a large sample and a substantial portion of participants tested positive for the coronavirus, it remains possible that we were not well powered to detect small changes in long-term mental health among the population sub-groups examined. Furthermore, it may be the case that COVID-19 is associated with increases in specific mental health symptoms, such as those associated with post-traumatic stress disorder, not assessed in the current study (Daly, MacLachlan, et al., 2021; Yuan et al., 2021). In addition, further examination of how trends in mental health symptoms as a result of contracting COVID-19 compare to trends following other illnesses (e.g. influenza and respiratory tract infection) is now needed (Taquet et al., 2021a, 2021b).

## Conclusions

Among a nationally representative sample of US adults, testing positive for COVID-19 is associated with an initial increase in psychological distress that diminishes quickly as symptoms subside. Although COVID-19 may not produce lasting psychological distress among the majority of the general population it remains possible that a minority may suffer longer-term mental health consequences.

## Data Availability

The research data are distributed by the USC Dornsife Center for Economic and Social Research and available at https://uasdata.usc.edu/index.php.

## Supplementary material

For supplementary material accompanying this paper visit http://dx.doi.org/10.1017/S003329172100324X. click here to view supplementary material
